# Expansion of tumor-infiltrating lymphocytes and their potential for application as adoptive cell transfer therapy in human breast cancer

**DOI:** 10.18632/oncotarget.23007

**Published:** 2017-12-06

**Authors:** Hee Jin Lee, Young-Ae Kim, Chan Kyu Sim, Sun-Hee Heo, In Hye Song, Hye Seon Park, Suk Young Park, Won Seon Bang, In Ah Park, Miseon Lee, Jung Hoon Lee, Yeon Sook Cho, Suhwan Chang, Jaeyun Jung, Jisun Kim, Sae Byul Lee, Sung Youl Kim, Myeong Sup Lee, Gyungyub Gong

**Affiliations:** ^1^ Department of Pathology, University of Ulsan College of Medicine, Asan Medical Center, Seoul, Korea; ^2^ Asan Center for Cancer Genome Discovery, Asan Institute for Life Sciences, University of Ulsan College of Medicine, Asan Medical Center, Seoul, Korea; ^3^ Lab of Molecular Immunology and Medicine, Department of Biomedical Sciences, University of Ulsan College of Medicine, Seoul, Korea; ^4^ Department of Biomedical Sciences, University of Ulsan College of Medicine, Seoul, Korea; ^5^ Department of Surgery, University of Ulsan College of Medicine, Asan Medical Center, Seoul, Korea; ^6^ R&D Center, GNSBio Co. Ltd., Gyeonggi-do, Korea

**Keywords:** breast cancer, tumor-infiltrating lymphocyte, adoptive cell transfer, memory T cell, function of TIL

## Abstract

Adoptive cell transfer (ACT) of *ex vivo* expanded tumor-infiltrating lymphocytes (TILs) has been successful in treating a considerable proportion of patients with metastatic melanoma. In addition, some patients with several other solid tumors were recently reported to have benefited clinically from such ACT. However, it remains unclear whether ACT using TILs is broadly applicable in breast cancer, the most common cancer in women. In this study, the utility of TILs as an ACT source in breast cancers was explored by deriving TILs from a large number of breast cancer samples and assessing their biological potentials. We successfully expanded TILs *ex vivo* under a standard TIL culture condition from over 100 breast cancer samples, including all breast cancer subtypes. We also found that the information about the percentage of TIL and presence of tertiary lymphoid structure in the tumor tissues could be useful for estimating the number of obtainable TILs after *ex vivo* culture. The *ex vivo* expanded TILs contained a considerable level of central memory phenotype T cells (about 20%), and a large proportion of TIL samples were reactive to autologous tumor cells *in vitro*. Furthermore, the *in vitro* tumor-reactive autologous TILs could also function *in vivo* in a xenograft mouse model implanted with the primary tumor tissue. Collectively, these results strongly indicate that ACT using *ex vivo* expanded autologous TILs is a feasible option in treating patients with breast cancer.

## INTRODUCTION

Breast cancer is the most common cancer in women, affecting about 12% worldwide, and causing more than 40,000 deaths per year in the United States alone [1, 2]. Breast cancer can be divided into subtypes according to the expression of hormone receptors (HRs; estrogen receptor (ER) and progesterone receptor (PR)) and human epidermal growth factor receptor 2 (HER2). Because breast cancer is such a heterogeneous disease, most therapeutic benefit usually comes from subtype-specific treatment [3]. Although survival rates after treatment have improved recently, largely owing to the advancement in neoadjuvant and adjuvant therapies [4], there are still a relatively large number of patients with treatment-resistant breast cancer [3, 5] for whom new therapeutic options are needed.

In recent years, cancer immunotherapy has emerged as an additional treatment alternative [6]. Popular cancer immunotherapeutic modalities include administration of monoclonal antibodies targeting immune checkpoints, tumor vaccination, and adoptive transfer of T cells engineered to have chimeric antigen receptors (CAR-T cells) or tumor-infiltrating lymphocytes (TILs), which target tumor cells [7]. Although not successful in all cases, a considerable proportion of patients treated with such immunotherapies have gained good clinical benefit, including complete remission for several years, which has popularized the approach as an anti-cancer modality [8, 9]. Adoptive cell transfer (ACT) using TILs (adoptive TIL therapy) is one such immunotherapy. This therapy is based on *ex vivo* expansion of TILs from patients with cancer and reinfusion of the TILs into the patients; it was originally developed for treating patients with advanced melanoma [8]. Impressively, objective response rates of over 50% were observed in patients with metastatic melanoma after adoptive TIL therapy, and the complete remission rate reached up to 24% [8, 10–12]. When adoptive TIL therapy was applied to other solid tumors, including those of the uterus, cervix, lung, and gastrointestinal tract, some patients also showed excellent clinical responses [9, 13–15]. These results imply that other solid cancers, such as breast cancer, could be appropriate targets for the application of ACT; however, in breast cancer, extensive TIL cultures and evaluation of the therapeutic potential of adoptive TIL therapy have not been reported, although TIL culture is reported to be possible in breast cancer [16]. Furthermore, although the clinical importance of long-lived memory T cell subsets in *ex vivo* expanded TILs is well established in a number of cancers, in breast cancer the composition of memory T cell subsets among fresh TILs directly derived from cancer tissues and *ex vivo* expanded TILs has never been described [8, 17–19].

In this study, we successfully expanded TILs from breast tumor samples from over 100 individuals and showed that the expanded TILs containing central memory phenotype T cells could be useful as an ACT source.

## RESULTS

### Successful TIL cultures are possible from all breast cancer subtypes

**Figure 1:**
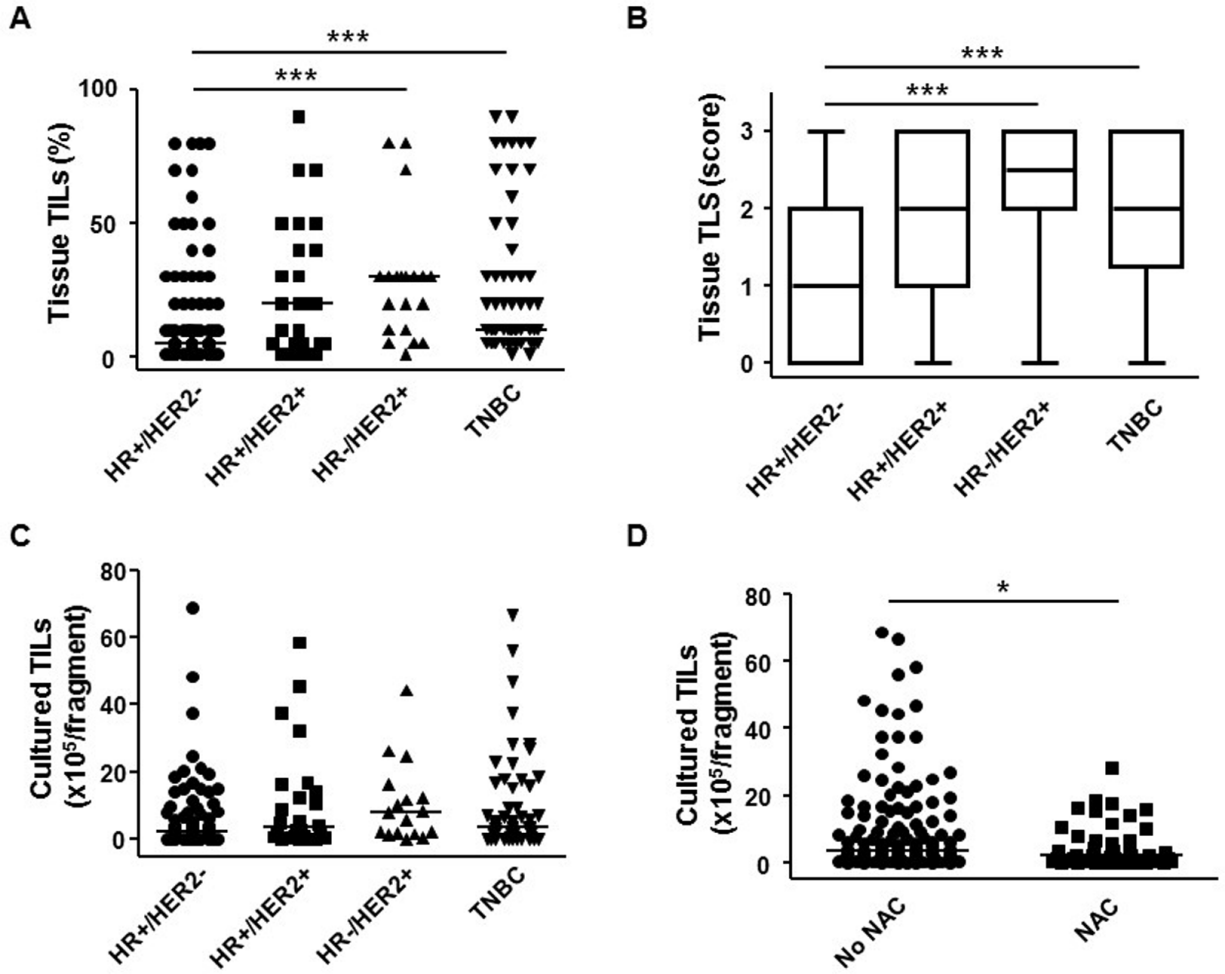
Tumor-infiltrating lymphocytes (TILs) from breast cancer tissues can be successfully expanded after 2 weeks’ *ex vivo* culture (**A**) Percentage (%) of TILs and (**B**) degree (score) of tertiary lymphoid structure (TLS) in HR^+^/HER2^−^ (*n* = 95), HR^+^/HER2^+^ (*n* = 26), HR^−^/HER2^+^ (*n* = 20), and TNBC (*n* = 56) breast cancer tissues. The degree of TLS was expressed as the following scores: 0, none; 1, little; 2, moderate; 3, abundant. (**C**) Number of TILs (per fragment) obtained after 2 weeks’ *ex vivo* culture from HR^+^/HER2^−^ (*n* = 83), HR^+^/HER2^+^ (*n* = 26), HR^−^/HER2^+^ (*n* = 17), and TNBC (*n* = 56) breast cancer tissues. (**D**) Number of TILs (per fragment) obtained after 2 weeks’ *ex vivo* culture from breast cancer tissues of patients treated with neoadjuvant chemotherapy (NAC) (*n* = 49) or without NAC (No NAC, *n* = 133). Kruskal–Wallis test and Mann–Whitney *U*-test were used for statistical analysis. ^*^p < 0.05, ^**^p < 0.01, ^***^p < 0.001. HR, hormone receptor; TNBC, triple-negative breast cancer.

Sources of TILs in *ex vivo* cultures are the cells within tumors and in the tertiary lymphoid structures (TLSs) in the tumor-adjacent tissue. Therefore, we first estimated the levels of TILs and TLSs in each subtype of breast cancer by analyzing hematoxylin and eosin (H&E)-stained sections of cancer tissues from 198 patients (Supplementary Table 1). The percentage of TILs and the degree of TLSs within HR^+^/HER2^−^ tissue were significantly lower than those in HR^−^/HER2^+^ or HR^−^/HER2^−^ triple-negative breast cancer (TNBC) subtypes (Figure 1A and 1B). However, the distributions of the percentage of TILs (median, 5%; range, 1–80% in HR^+^/HER2^−^; median, 20%; range, 1–90% in HR^+^/HER2^+^; median, 30%; range, 1–80% in HR^−^/HER2^+^; median, 10%; range, 1–90% in TNBC) and the degree of TLS (median score 1 in HR^+^/HER2^−^; median 2 in HR^+^/HER2^+^; median 2.5 in HR^−^/HER2^+^; median 2 in TNBC; and score range 0–3 for all subtypes) within each subtype were quite broad (Figure 1A and 1B), indicating that the subtype itself cannot provide a good indication of the levels of TILs or TLS.

To apply adoptive TIL therapy to cancer treatment, TILs from tumors should first be expanded. To expand TILs *ex vivo*, tumor samples (on average 63 tumor fragments derived from each tumor of on average 6 mm in diameter and 2–3 mm thickness) from 187 patients with breast cancer of all subtypes were cultured under a standard initial TIL culture condition for 2 weeks (Materials and methods; Supplementary Table 2). Except in five cases, we were able to obtain >1.0 × 10^3^ TILs per tumor fragment; the majority of cases (129/182) yielded >1.0 × 10^5^ TILs from each fragment (Supplementary Table 2). The number of TILs obtained from each breast cancer subtype was not significantly different among subtypes (median, 2.0 × 10^5^; range, 0.03–68.8 × 10^5^ in HR^+^/HER2^−^; median, 3.6 × 10^5^; range, 0.01–58.5 × 10^5^ in HR^+^/HER2^+^; median, 4.8 × 10^5^; range, 0.03–44.5 × 10^5^ in HR^−^/HER2^+^; median, 3.3 × 10^5^; range, 0.01–66.7 × 10^5^ in TNBC; Figure 1C). These results indicate that TILs can be stably obtained from the majority of breast cancers, regardless of subtype. Our samples were derived from patients treated both with and without neoadjuvant chemotherapy (NAC and No NAC, respectively; Supplementary Tables 1 and 2). After the initial 2 week culture of cells from both types of patient samples, the number of TILs obtained from NAC samples was significantly lower (4.7 ± 6.5 × 10^5^ *vs.* 8.9 ± 13.9 × 10^5^; *p* < 0.05) than that obtained from No NAC samples, indicating that chemotherapy reduced the mass of the tumor and/or number of TILs, but the degree of reduction was only slight (Figure 1D).

### TIL and TLS levels in tumor tissues provide a good indication of the TIL number obtainable after *ex vivo* culture

**Figure 2:**
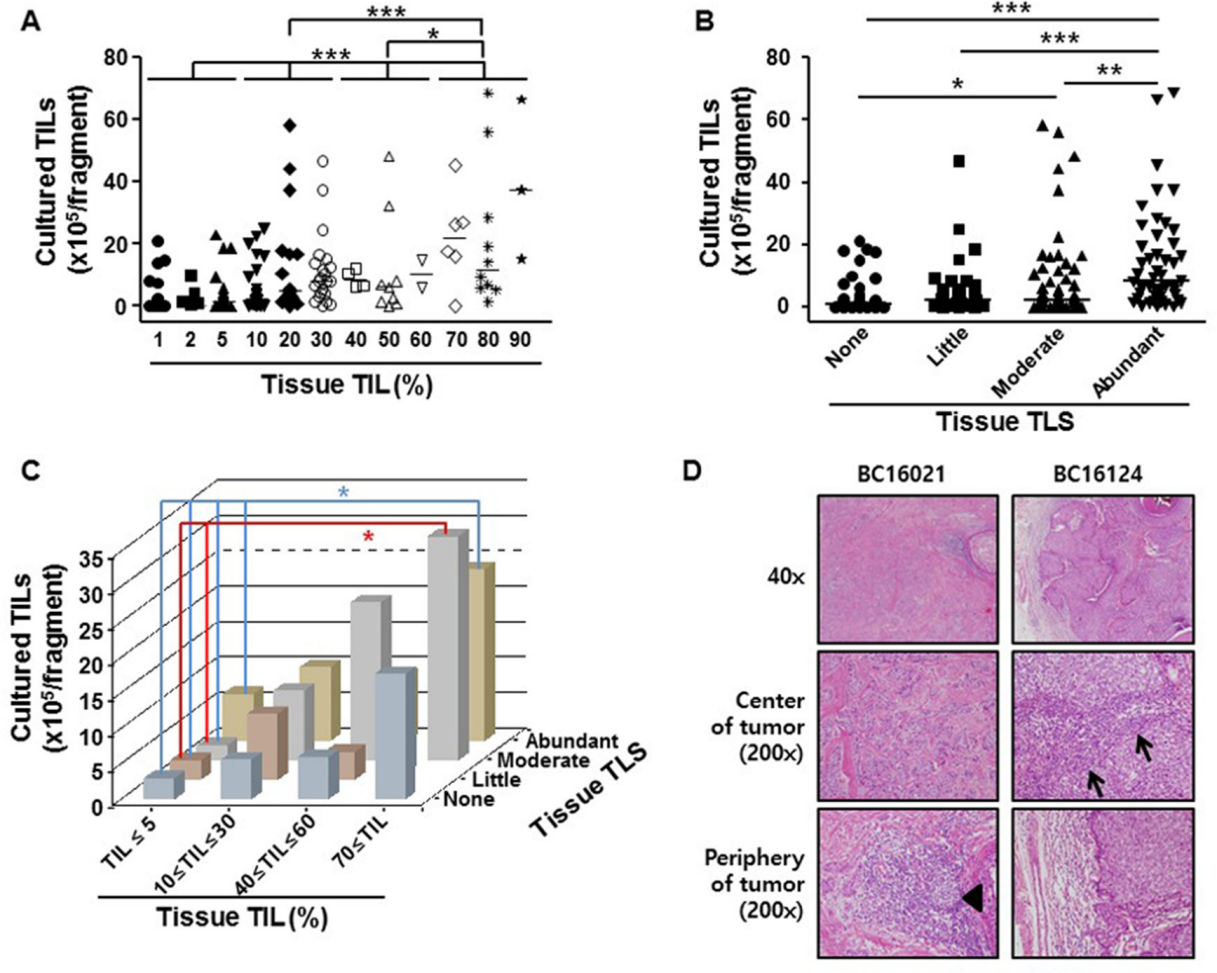
Correlation between the number of 2 week TILs and TIL percentage or TLS degree in breast cancer tissues (**A**) The number of TILs per fragment (*y*-axis) obtained after 2 weeks’ *ex vivo* culture from breast cancer tissues was plotted against histologically determined tissue TIL percentage (*x*-axis): Tissue TILs: 1% (*n* = 29), 2% (*n* = 5), 5% (*n* = 45), 10% (*n* = 26), 20% (*n* = 19), 30% (*n* = 22), 40% (*n* = 4), 50% (*n* = 9), 60% (*n* = 2), 70% (*n* = 6), 80% (*n* = 10), and 90% (*n* = 3). Tumor samples were grouped as follows for statistical analysis: TILs ≤5%, 10%≤ TIL ≤30%, 40%≤ TIL ≤60%, or 70% ≤ TIL. (**B**) The number of TILs per fragment (*y*-axis) obtained after the 2 week culture was plotted against tissue TLS degree (*x*-axis) grouped as follows: Tissue TLS degree, none (*n* = 42), little (*n* = 35), moderate (*n* = 56), and abundant (*n* = 47). (**C**) The average number of TILs per fragment (*z*-axis) obtained after the 2 week culture was plotted in the space defined by both percentage of tissue TILs (*x*-axis) and TLS degree (*y*-axis) in the same sample: 1%≤ tissue TILs ≤5% and TLS none (*n* = 36); 1%≤ tissue TILs ≤5% and TLS little (*n* = 19); 1%≤ tissue TILs ≤5% and TLS moderate (*n* = 19); 1%≤ tissue TILs ≤5% and TLS abundant (*n* = 5); 10%≤ tissue TILs ≤30% and TLS none (*n* = 4); 10%≤ tissue TILs ≤30% and TLS little (*n* = 14); 10%≤ tissue TILs ≤30% and TLS moderate (*n* = 32); 10%≤ tissue TILs ≤30% and TLS abundant (*n* = 17); 40%≤ tissue TILs ≤60% and TLS none (*n* = 1); 40%≤ tissue TILs ≤60% and TLS little (*n* = 2); 40%≤ tissue TILs ≤60% and TLS moderate (*n* = 3); 40%≤ tissue TILs ≤60% and TLS abundant (*n* = 9); 70%≤ tissue TILs and TLS none (*n* = 1); 70%≤ tissue TILs and TLS little (*n* = 0); 70%≤ tissue TILs and TLS moderate (*n* = 2); and 70%≤ tissue TILs and TLS abundant (*n* = 16). Kruskal–Wallis test and Mann–Whitney *U*-test were used for statistical analysis. ^*^p < 0.05, ^**^p < 0.01, ^***^p < 0.001. (**D**) Representative examples of H&E sections showing low numbers of TILs (5%) and little TLS (left panels: sample BC16021, 18.7 **×** 10^5^ TILs/fragment obtained after 2 weeks’ culture), and high numbers of TILs (70%) and no TLS (right panels: sample BC16124, 17.7 **×** 10^5^ TILs/fragment obtained after culture). Arrows indicate TILs, and the arrowhead indicates TLS.

To explore whether analysis of the cancer tissue before culture could be useful for predicting the obtainable TIL numbers after the initial 2 week culture, the percentage of TILs or degree of TLS was estimated histologically from cancer tissue sections (Materials and methods). The group with ≤5% TILs observed in the tumor tissue yielded a significantly lower number of cultured TILs than those groups (10% ≤ TIL ≤ 30%, 40% ≤ TIL ≤ 60%, or 70% ≤ TIL) with a higher percentage of TILs observed in the tissue, while the tumor group with 70% ≤ TILs in tissues gave significantly higher numbers of cultured TILs than those groups with a lower percentage of TILs in tissues (Figure 2A, Supplementary Table 2). Similarly, tumors with abundant TLSs yielded significantly higher numbers of cultured TILs than those graded as having ‘none’, ‘little’, or ‘moderate’ TLS (Figure 2B, Supplementary Table 2). Consistently, there was a significant, positive correlation between the number of obtained TILs and the percentage of TILs in cancer tissues (correlation coefficient *ρ* = 0.527, *p* < 0.001), and between the number of obtained TILs and TLS degree (correlation coefficient *ρ* = 0.392, *p* < 0.001). Since both TIL percentage and TLS degree in tissues can affect the number of TILs obtained after 2 week culture, the number of obtained TILs was analyzed by simultaneously taking into account the TIL percentage and TLS degree for the same sample. As expected, the tumor group with the highest percentage of TILs observed in the tissue (≥70%) and abundant TLSs yielded a much higher number of TILs after the 2 week culture than the groups with a low percentage of TILs (≤5%) and none, little, moderate, or abundant TLSs, while the tumor group with the highest percentage of TILs (≥70%) and a moderate degree of TLS yielded a much higher number of TILs after 2 weeks’ culture than those groups with a low percentage of TILs (≤5%) and a little or moderate degree of TLS (Figure 2C and 2D).

The minimum number of TILs required for ACT in a clinical setting is around 1 × 10^10^ per patient [20–22]. The standard rapid expansion protocol (REP), a second round of culture after the 2 week initial culture, can expand TILs by 1,000-fold [23], and the usual obtainable tumor samples (≥8 mm in diameter and 2–3 mm thickness) can provide more than 100 fragments; thus if >1.0 × 10^5^ TILs are obtainable from each tumor fragment after only the initial 2 weeks’ culture, sufficient TILs for ACT in an autologous patient would be expected after the standard REP. When the distribution of the number of TILs obtained from one tumor fragment after 2 weeks’ culture was analyzed by simultaneously taking into account the TIL percentage and TLS degree for the same sample, interestingly, the majority of tumor fragments with ≥10% TILs (regardless of TLS level; in 87/102 cases) or with abundant TLS (regardless of TIL percentage; in 44/48 cases) yielded more than 1.0 × 10^5^ TILs after 2 weeks’ culture (Figure 2C, Supplementary Table 2). These results suggest that TIL percentage and TLS degree in tumor tissues are very informative for predicting the number of easily obtainable TILs, which would be important for planning a schedule for ACT with *ex vivo* expanded TILs.

### Memory phenotype T cells are the major subset after TIL culture

**Figure 3:**
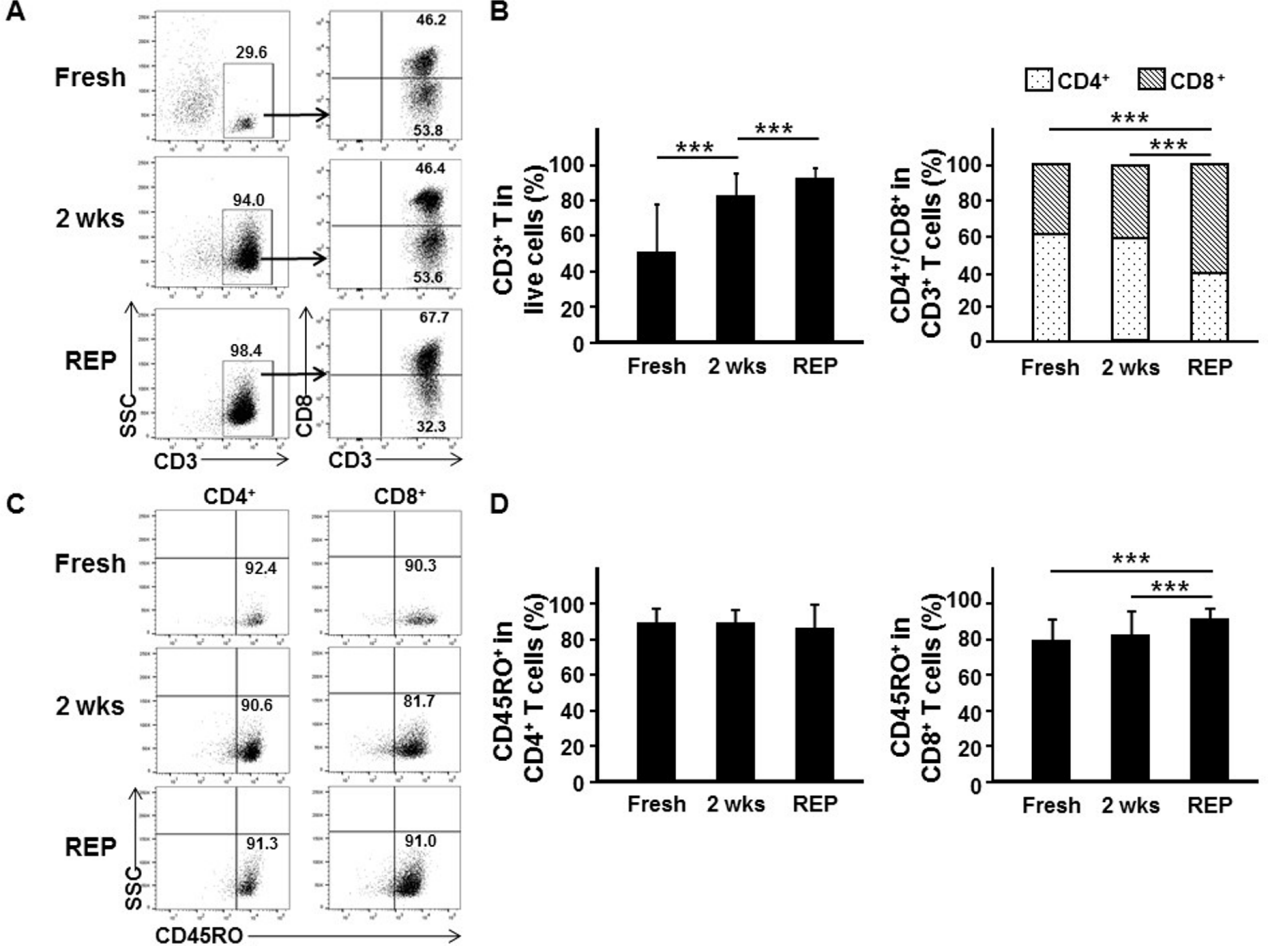
*Ex vivo* TIL culture enriches memory phenotype CD8^+^ T cells Analysis of T cells and their subsets (**A**, **B**), and memory phenotype T cells (**C**, **D**) in TILs. Single cells directly derived from fresh breast cancer tissues (fresh, *n* = 33), TILs obtained after 2 weeks’ *ex vivo* culture of breast cancer tissue fragments (2 wks, *n* = 132), and post-REP TILs (REP, *n* = 45) were analyzed by flow cytometry. (A) Representative FACS data showing percentage of T cells (CD3^+^) among live cells, percentage of CD4^+^ T cells (CD3^+^CD8^−^) and of CD8^+^ (CD3^+^CD8^+^) T cells among T cells, and (C) percentages of memory phenotype CD4^+^ T cells (CD3^+^CD8^−^CD45RO^+^) and CD8^+^ T cells (CD3^+^CD8^+^CD45RO^+^) among CD4^+^ T cells (CD3^+^CD8^−^) and CD8^+^ T cells (CD3^+^CD8^+^), respectively. (B, D) FACS results expressed as the mean ± SD summarized as histograms. Kruskal–Wallis test and Mann–Whitney *U*-test were employed for statistical analysis. ^***^p < 0.001.

Production of large numbers of TILs is necessary for ACT therapy, and therefore the REP is usually required. When such expansion was performed with the TILs obtained after the initial 2 weeks’ culture, most of the initial TILs expanded further in number (2,053 ± 290-fold; Supplementary Table 3). Since it is T cells that are mainly responsible for tumor cell control [24, 25], we assessed the proportion of T cells in TILs by fluorescence-activated cell sorting (FACS) before and after culture (see Supplementary Figure 1 for gating strategy). The proportion of CD3^+^ T cells was markedly increased after culturing fresh TILs (directly derived from the tumor tissues, 49.7% ± 27.6%) for 2-weeks (81.5% ± 12.8%) and REP (91.6% ± 6.9%; Figure 3A and 3B). However, the proportion of other major immune cells such as B cells and myeloid cells (except the proportion of natural killer cells, which increased slightly during the initial 2 weeks’ culture, but decreased later) drastically decreased after the *ex vivo* culture (Supplementary Figure 2).The proportion of CD8^+^ T cells among CD3^+^ TILs was also increased after the REP (fresh (39.0% ± 14.3%), after 2 weeks’ culture (41.5% ± 20.6%), and post-REP (60.8% ± 24.7%); Figure 3A and 3B). For ACT to be effective, tumor-reactive memory T cells should be present in the expanded TILs. Thus, the proportion of T cells expressing CD45RO (broadly defined as a memory T cell marker) [26, 27] in fresh and cultured TILs was assessed (see Supplementary Figure 1 for gating strategy). The majority (≥79.0%) of T cells were CD45RO^+^ memory T cells, regardless of the *ex vivo* cultures, indicating that memory T cells present in the tissues readily expanded *ex vivo* (Figure 3C and 3D). The proportion of CD45RO^+^ cells among CD4^+^ T cells was not significantly changed after *ex vivo* culture (fresh TILs (88.5% ± 9.0%), after 2 weeks’ culture (88.9% ± 7.8%), and post-REP (85.7% ± 14.3%)), while the proportion of CD45RO^+^ cells among CD8^+^ T cells slightly increased in post-REP TILs (fresh TILs (79.1% ± 12.4%), after 2 weeks’ culture (81.9% ± 14.0%), and post-REP (90.8% ± 6.9%); Figure 3C and 3D).

### Central memory phenotype T cells -in cancer tissues are readily expanded after TIL culture

**Figure 4:**
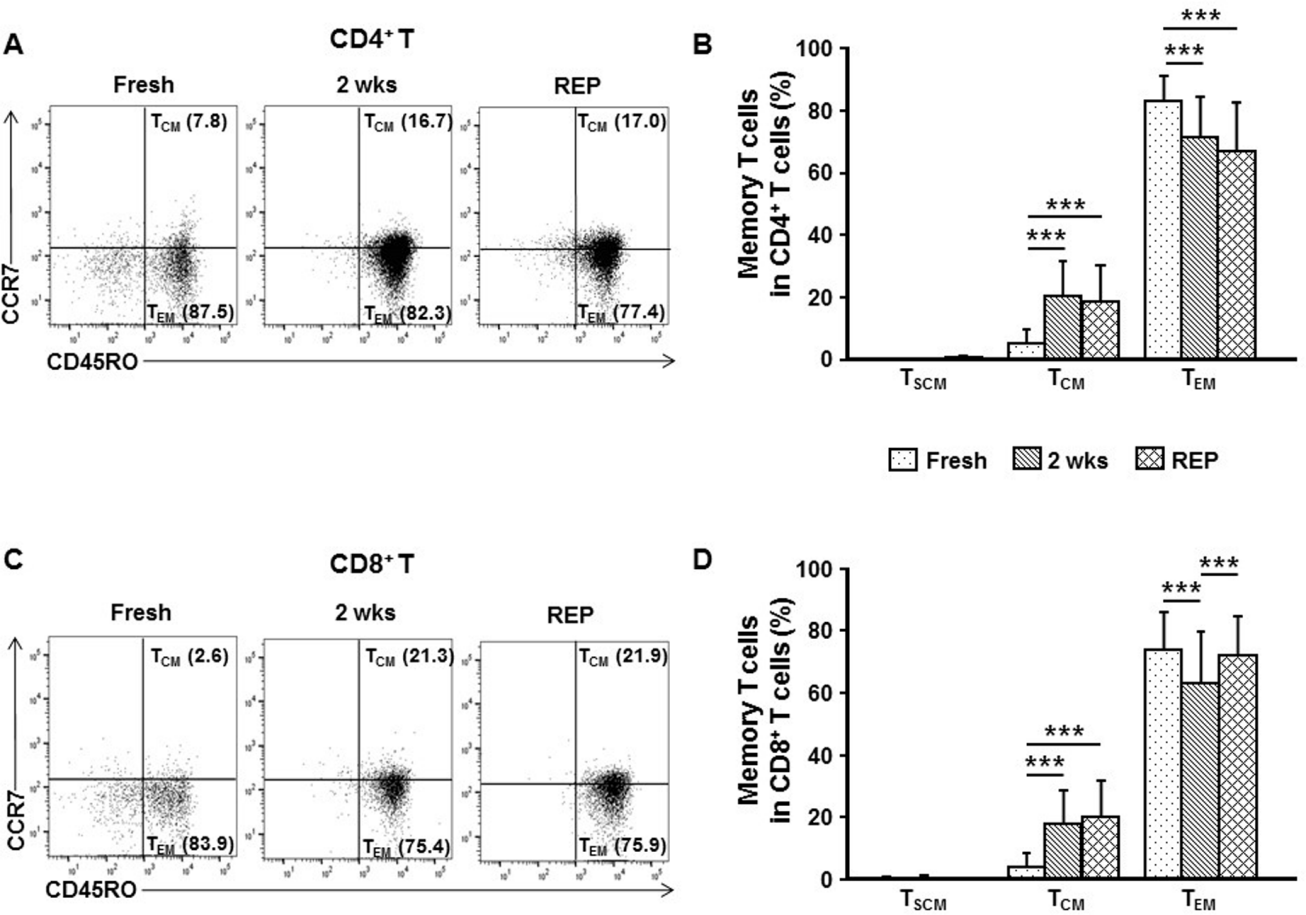
*Ex vivo* TIL culture does not compromise the proportion of the central memory phenotype T cell subset Memory CD4^+^ (**A**, **B**) and CD8^+^ (**C**, **D**) T cell subpopulations among single cells directly derived from fresh breast cancer tissue (fresh, *n* = 33), those among TILs obtained after the 2 week *ex vivo* culture of breast cancer tissue fragments (2 wks, *n* = 132), and those among post-REP TILs (REP, *n* = 45) were analyzed by flow cytometry. Representative FACS data showing percentage of memory stem T cells (T_SCM_, CD45RO^−^CCR7^+^CD62L^+^CD95^+^), central memory phenotype T cells (T_CM_, CD45RO^+^CCR7^+^), or effector memory T cells (T_EM_, CD45RO^+^CCR7^−^) among CD4^+^ (A) and CD8^+^ (C) T cells. (B, D) FACS results expressed as the mean ± SD summarized as histograms. Kruskal–Wallis test and Mann–Whitney *U*-test were employed for the statistical analysis. ^***^p < 0.001.

Since expansion of broadly defined memory phenotype T cells in cultured TILs was observed, we then analyzed the composition of memory T cell subsets in fresh, initial 2 week cultured, and post-REP TILs by FACS (see Supplementary Figure 3 for gating strategy). The majority of the CD4^+^ and CD8^+^ T cells were effector memory T cells (T_EM_, defined as CD45RO^+^CCR7^−^, which are expected to be short-lived *in vivo*) among the fresh TILs (83.0% ± 7.8% for CD4^+^ T cells; 73.5% ± 12.5% for CD8^+^ T cells), the initial 2 week cultured TILs (71.2% ± 13.4% for CD4^+^ T cells; 62.9% ± 16.5% for CD8^+^ T cells), and the post-REP TILs (66.9% ± 15.5% for CD4^+^ T cells; 71.7% ± 12.9% for CD8^+^ T cells; Figure 4) [26, 27]. Memory stem T cells (T_SCM_, defined as CD45RO^−^CCR7^+^CD62L^+^CD95^+^) were mostly absent in the fresh TILs (0.1% ± 0.3% for CD4^+^ T cells; 0.3% ± 0.7% for CD8^+^ T cells) as well as in the initial 2 week cultured TILs (0.1% ± 0.3% for CD4^+^ T cells; 0.4% ± 1.0% for CD8^+^ T cells) and post-REP TILs (0.4% ± 0.7% for CD4^+^ T cells; 0.2% ± 0.4% for CD8^+^ T cells; Figure 4). The proportion of central memory T cells (T_CM_, defined as CD45RO^+^CCR7^+^) was relatively low in fresh TILs (5.1% ± 4.7% for CD4^+^ T cells; 3.8% ± 4.5% for CD8^+^ T cells), increased after the initial 2 week culture (19.9% ± 11.6% for CD4^+^ T cells; 17.9% ± 10.4% for CD8^+^ T cells), and did not change significantly during the REP (18.2% ± 12.0% for CD4^+^ T cells; 19.8% ± 12.0% for CD8^+^ T cells; Figure 4). These results indicate that long-term TIL culture under the current conditions is possible without losing the potentially long-living T_CM_.

### TILs obtained after the REP are functional *in vitro*

**Figure 5:**
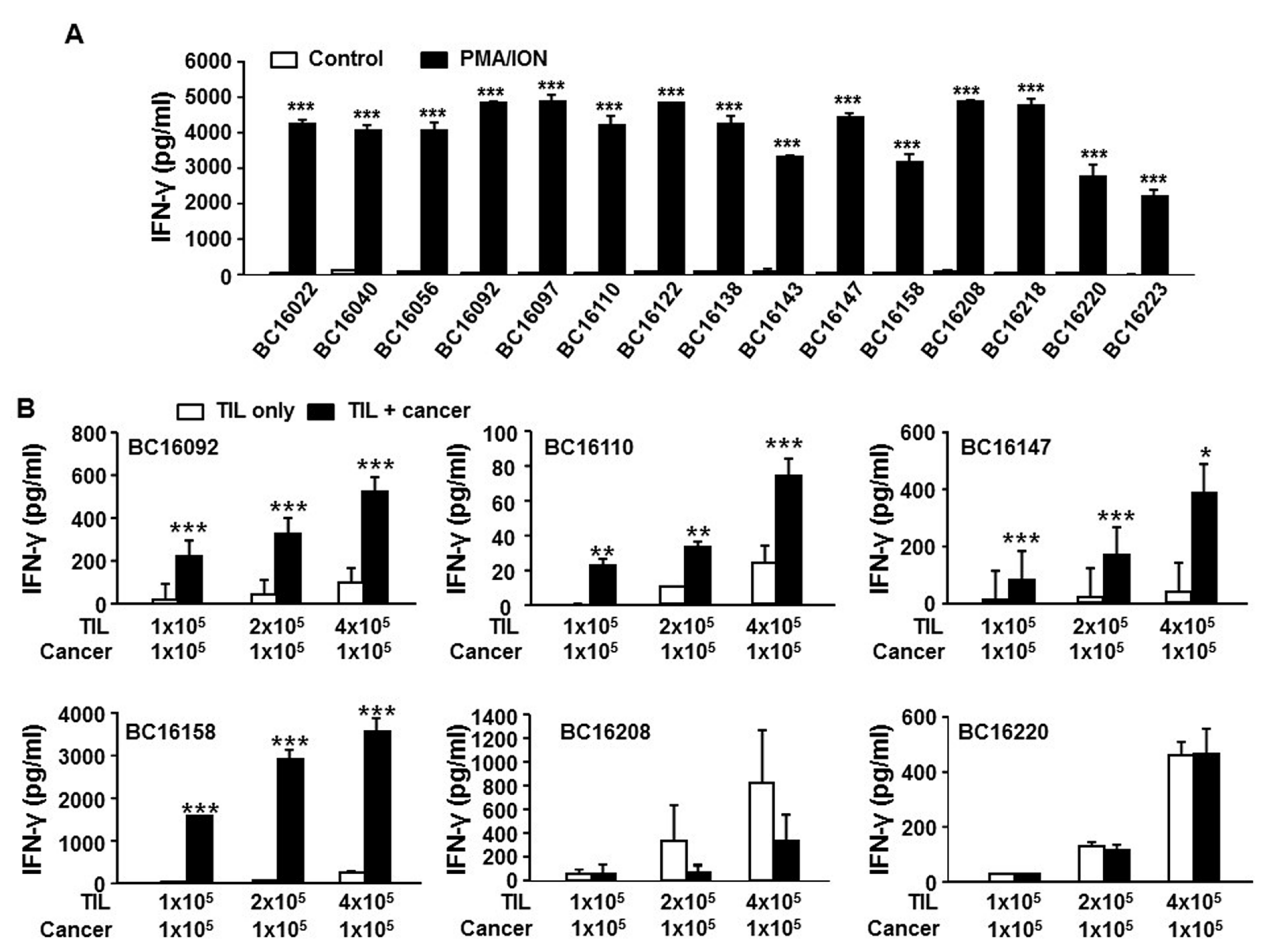
Post-REP TILs are functional and retain reactivity towards autologous primary tumor cells cultured *in vitro* Two week cultured TILs were further expanded for 2 weeks under a standard REP condition and tested for functional activity *in vitro*. (**A**) PMA/ION response: 1 × 10^5^ post-REP TILs (from each of 15 randomly selected samples; sample IDs are shown) were seeded in 96-well plates and treated with or without PMA (32.4 nM)/ION (1 μg/ml). After 24 h, cell culture supernatants were collected and the IFNγ level was measured by ELISA (*n* = 3 or 4 per group). Open bars indicate no treatment (control), and filled bars indicate PMA/ION treatment (PMA/ION). (**B**) The reactivity of TILs to autologous primary cancer cells. Post-REP TILs from six selected samples were co-cultured with (filled bar) or without (open bar) the autologous primary tumor cells (1 × 10^5^) at an effector:target cell ratio of 1:1, 2:1, or 4:1. After 24 h, cell culture supernatants were collected and the IFNγ level was measured by ELISA (*n* = 3 or 4 per group). A non-paired Student’s *t*-test was performed. ^*^p < 0.05, ^**^p < 0.01, ^***^p < 0.001.

To determine if the post-REP TILs are potentially functional, we performed *in vitro* stimulation with phorbol myristate acetate (PMA) and ionomycin (ION), followed by assessment of interferon-γ (IFNγ) production on 15 randomly selected post-REP TILs that either had accompanying autologous primary tumor cells or patient-derived xenograft (PDX) mice implanted with autologous primary tumor tissues. All the TILs tested produced IFNγ at a considerable level (>2,000 pg/mL) upon stimulation, indicating that post-REP TILs retain functional activity (Figure 5A). To determine the reactivity of post-REP TILs towards the primary tumor, the TILs were co-cultured with available autologous primary tumor cells and IFNγ production was determined by ELISA. IFNγ production from 4/6 post-REP TIL samples was increased in the presence of autologous tumor cells (Figure 5B). Furthermore, increased IFNγ production from post-REP TILs against autologous primary tumor cells was decreased by addition of anti-MHC I blocking antibody but not by addition of anti-MHC II blocking antibody (Supplementary Figure 4), indicating that the response of the TILs to autologous tumor cells is MHC I-restricted, but not MHC II-restricted. Consistently, all autologous tumor cells eliciting TIL reactivity expressed MHC I on the cell surface, while those tumors against which TILs were unreactive did not (Supplementary Figure 5). Furthermore, the expression of MHC II on the tumor cell surface was minor, if expressed (Supplementary Figure 5) and both tumor-reactive post-REP TILs and -non-reactive post-REP TILs showed similar cell composition (almost all T cells in both; Supplementary Figure 6). Therefore, as expected, self-MHC I recognition by post-REP T cells seems to be the most important parameter determining TIL reactivity to tumor cells. This result suggests that ACT would be possible for patients with *in vitro* tumor-reactive TILs but that not all patients could benefit from ACT.

### Post-REP TILs can be functional in the PDX mouse model

**Figure 6:**
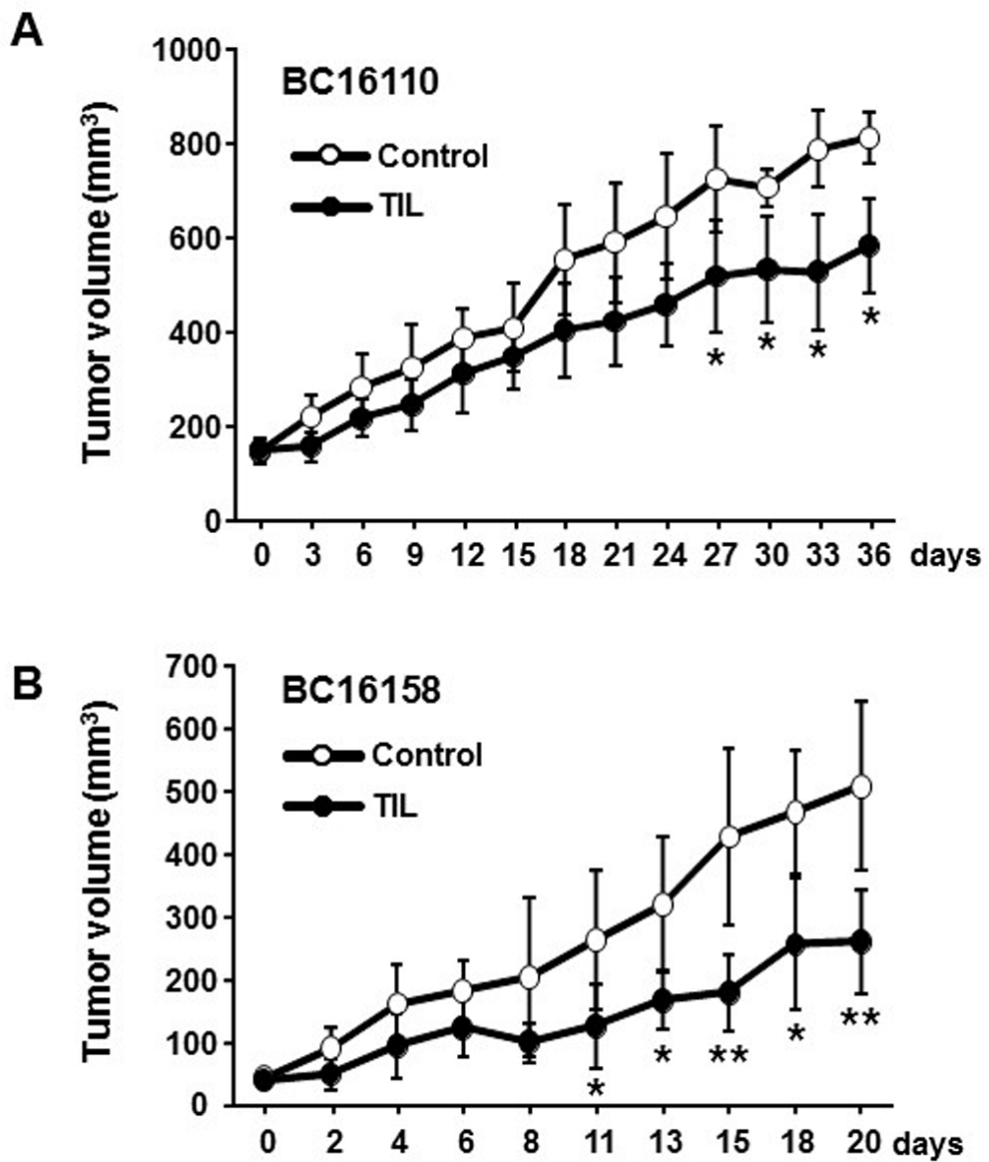
*In vitro* tumor-reactive post-REP TILs are functional in a PDX mouse model *in vivo* PDX mice were created by implanting tumor tissues derived from each of two patient samples (from a lymph node with metastatic breast cancer, sample BC16110 (**A**), and from breast, sample BC16158 (**B**)). When tumors in the PDX mice were palpable, 1 × 10^7^ post-REP TILs (TIL group, *n* = 4 for BC16110 and *n* = 5 for BC16158) or PBS (control group, *n* = 4 for BC16110 and *n* = 5 for BC16158) was administered into the tail vein, and IL-2 (200,000 IU/mice) and IL-15 (1 μg/mice) were administered simultaneously into the peritoneal cavity of the mice in both groups. Then, IL-2 (200,000 IU/mice) was administered for the following 2 days (total of three times) and IL-15 (1 μg/mice) was administered every other day for 8 days (total of five times). After administration of TILs (day 0), tumor size was measured using calipers. Tumor volumes were calculated using the formula ½ × length × (width)^2^ and expressed as the mean ± SD. A non-paired Student’s *t*-test was performed. ^*^p < 0.05, ^**^p < 0.01.

To determine if the post-REP TILs with reactivity towards autologous primary tumor cells *in vitro* can also be effective *in vivo*, adoptive transfer of tumor-reactive post-REP TILs into PDX mice successfully implanted with autologous primary tumor tissue was performed and the tumor size was measured over time. The autologous tumors in the PDX mice administered with 1.0 × 10^7^ post-REP TILs grew much slower than those in mice not administered with TILs (Figure 6). This result strongly indicates that TILs from breast cancers could be exploited as an ACT source.

## DISCUSSION

In this study, we showed that TILs can be obtained from all the breast cancer subtypes and that information on the TIL percentage and TLS degree in tumor samples is useful for estimating the number of obtainable TILs after an initial 2 week *ex vivo* culture. In addition, we showed that such expanded and post-REP TILs contained a considerable level of T_CM_, that a large proportion of post-REP TIL samples were reactive to autologous tumor cells *in vitro*, and that these *in vitro* tumor-reactive post-REP TILs could function in an *in vivo* PDX model. To the best of our knowledge, this is the largest study describing successful *ex vivo* expansion of TILs and evaluating their potential as an ACT source in breast cancer.

Successful TIL expansion from several cancer types has been reported, including melanoma, colorectal cancer, renal cell carcinoma, and pancreatic cancer [20, 25, 28, 29]. Regarding breast cancer, a few reports have described initial TIL cultures; however, the case numbers in these studies were small (<20), and reactivity of TILs to autologous tumors and the proportion with memory T cell phenotype, critical parameters for predicting therapeutic efficacy, were not described, making it difficult to ascertain the utility of ACT [16, 24]. In this study, we successfully expanded a large number of TILs (from more than 100 cancer samples), which were potentially usable for ACT in a clinical setting after a standard REP was performed. We consider the essential TIL number after the initial 2 week culture to be ≥1.0 × 10^7^ cells, which, combined with the over 1,000-fold expansion of TILs possible under a standard REP condition, will provide the ≥1.0 × 10^10^ TILs per patient known to be necessary for use in ACT [20–22]. In this study, the success rate in producing this essential number was about 70% (129/182), determined by considering that usual tumor samples (≥ 8 mm in diameter and 2–3 mm thickness) can provide over 100 fragments, and the number of cases that yielded more than 1.0 × 10^5^ TILs from one fragment after the initial 2 week culture was 129 out of 182. This result indicates that a sufficient number of TILs can be obtained from the majority of breast cancer samples. The success rate reported for *ex vivo* expansion of TILs from other cancer studies is around 60–85% [11, 24, 30], which is not different from that obtained in the present study, indicating that breast cancers are not different from other solid tumors in terms of TIL expandability.

We also cultured TILs from many fewer tumor fragments (<30 fragments) in 32 cases because of the limited size of the available tumor tissue (<4 mm in diameter). While 22 samples yielded >1 × 10^5^ cells per tumor fragment (Supplementary Table 2), the majority of these cases (20/32, 62.5%) had too few fragments to expand TILs to the clinically sufficient >1 × 10^7^ cells, indicating that tumor tissues <4 mm in diameter would be impractical for use in ACT. In cases of samples that yielded <1.0 × 10^7^ cells after the initial 2 week culture, a more extended initial TIL culture period may help increase cell number. Indeed, in some reports, TILs were initially cultured for 4 weeks to obtain at least 3 × 10^7^ cells from melanoma tissue fragments [21]. Thus, with this possibility in mind, the success rate of obtaining a sufficient number of TILs after *ex vivo* culture is likely to be much higher than that obtained in this study, and considering that the proportion of T_CM_ did not decrease significantly after initial TIL and REP cultures, longer-cultured cells are likely to be capable of surviving *in vivo* and functioning similarly to shorter-cultured cells. Furthermore, since the *ex vivo* expansion of TILs from TNBC sample was recently reported to be enhanced in the presence of 4–1BB agonistic antibody (against CD137, a TNF receptor superfamily member 9), it is possible that this approach could enhance TIL expansion from other breast tumor types [31].

It would be very useful for planning ACT if routine histological analysis, performed on tumor tissues by pathologists, could give good information about the number of TILs likely to be expanded *ex vivo*. Therefore, in the present study, the number of TILs obtained after the initial 2 week culture was analyzed together with tissue parameters related to TIL number such as the percentage of TILs and the degree of TLS observed in the tissue sections. As expected, predominantly, the samples with a higher percentage of TILs observed in the tissue yielded more cultured TILs. Interestingly, when we considered TIL percentage and TLS level together in the same tissue sample, even samples with a low percentage of TILs inside the invasive tumor border could yield sufficient numbers of expanded TILs if they contained abundant TLSs beyond the invasive tumor margin. This result shows that TLSs from solid tumors contribute significantly to the number of TILs obtained after *ex vivo* expansion of TILs. In addition, this indicates that histological analysis for assessing TIL percentage and TLS degree in tumor tissues could provide useful information for designing an ACT plan.

As expected, in this study, *ex vivo* TIL culture enriched T cells. Among T cells, the proportions of CD4^+^ and CD8^+^ T cells were comparable in fresh TILs and in the initial 2 week cultured TILs, and the proportion of CD8^+^ T cells increased after the REP culture. Since the major anti-cancer effector T cells are known to be CD8^+^ T cells [31, 32], and in the present study we found CD8^+^ T cells to account for a major portion (∼60%) of the post-REP TILs, we speculate that CD8^+^ T cells would have played a major role in mediating the anti-tumor reactivity we observed *in vitro* and *in vivo* after ACT in the PDX model. However, since CD4^+^ T cells were also present as a considerable fraction (∼40%) in the post-REP T cells and adoptive transfer of CD4^+^ T cells is reported to be capable of managing various cancers including cholangiocarcinoma and melanoma successfully [13, 33], we cannot exclude a significant contribution by the CD4^+^ T cells to the anti-tumor immune response. For ACT to be effective, memory T cells with an extended viability are considered to be critical [27]. Indeed, existence of memory T cell subsets with prolonged viability in *ex vivo* expanded TILs has been shown to be important for therapeutic efficacy in mouse and human studies [27, 34, 35]. In the current study on breast cancer, we observed that the proportion of T_CM_ did not decline significantly during *ex vivo* TIL cultures, and the proportion even increased after the initiation of *ex vivo* culture. In addition, the proportion of T_CM_ was not low (about 20%) after TIL culture. These results together suggest that T_CM_ would play a major role in tumor control in adoptive TIL therapy applied in breast cancer. The preferential increase of the T_CM_ population after the initiation of *ex vivo* TIL culture could be caused by the liberation from the suppression of T_CM_ proliferation/survival/differentiation induced by the immunosuppressive tumor environment [36, 37]. TGF-β is likely to be one of the main factors responsible for this suppressive environment, as it can inhibit clonal expansion of memory CD8^+^ T cells and inhibit T_CM_ differentiation [36, 38].

All the post-REP TILs tested showed a good response to PMA/ION, indicating that TILs are functionally active after *ex vivo* culture. However, not all the TIL samples co-cultured with autologous primary tumor cells were reactive to the tumor cells. These results indicate that TILs from certain samples cannot recognize the autologous tumor. We presume that one of the main reasons for the unresponsiveness of TILs to certain autologous tumors is downregulation of MHC I molecules on the tumor cells, known to occur in some cancers [39], because we observed that TILs responded well to autologous, cultured primary tumor cells expressing MHC I on the cell surface but not to those without MHC I (Supplementary Figures 4 and 5). When post-REP TILs reactive to autologous tumors *in vitro* were administered to PDX mice bearing autologous primary tumor tissues, the tumor growth was retarded. Although, because of a low PDX implantation rate, we could test this in only two cases (one bearing breast-derived, the other metastatic lymph node tumor tissue), we obtained reasonable positive responses from both cases. Therefore, TILs reactive to autologous tumors *in vitro* were apparently effective *in vivo* as well. Taken together, these results strongly suggest that ACT of post-REP TILs into patients with breast cancer could benefit those patients whose TILs show good reactivity to autologous tumor cells *in vitro*.

Among the cancer samples analyzed, we included samples derived from patients treated systemically with neoadjuvants. Although these neoadjuvant-treated samples yielded a slightly smaller number of TILs after *ex vivo* culture, the basic features, such as memory phenotype and expansion levels, were not different from those of samples from patients not treated with neoadjuvants. Since patients containing residual tumors after systemic neoadjuvant therapy, especially those with TNBC, are prone to cancer recurrence after the therapy, such high-risk patients might benefit from adoptive TIL transfer [40]. In addition, because we were able to culture a comparable number of TILs (>1.0 × 10^5^ TILs) from metastatic sites of breast cancer (Supplementary Table 2 and data not shown), patients with distant metastasis could particularly benefit from ACT with *ex vivo* expanded TILs.

In this study, we successfully expanded TILs from over 100 breast cancer samples including all the breast cancer subtypes and showed that the *ex vivo* expanded TILs contained a significant proportion of T_CM_ cells and a large proportion of TIL samples were reactive to autologous tumors. Together, these results indicate that ACT using *ex vivo* expanded TILs is feasible in breast cancer. Thus, in the future, ACT of TILs derived from breast cancers could become a good treatment alternative for certain patients with a severe disease burden.

## MATERIALS AND METHODS

### Patients and histological evaluation of breast cancer tissues

Breast cancer tissue obtained during surgery with or without neoadjuvant systemic chemotherapy was collected from 198 female patients after obtaining informed consent before surgery in 2016. All breast cancer tissue was derived from the breast except two cases from lymph nodes with metastasis (Supplementary Tables 1 and 2). This study was approved by the Institutional Review Board of Asan Medical Center (IRB#2015–0438).

The H&E-stained slides were reviewed by two pathologists (H.J.L. and G.G.), and the percentage of TILs was determined. Percentage of TILs was defined as the mean percentage of the stroma with invasive carcinoma infiltrated by lymphocytes and plasma cells. Presence of TILs was estimated in 10% increments, or if less than 10% of the stroma was infiltrated, as 1%, 2%, or 5% [41, 42]. The degree of TLS in tumor-adjacent tissue (scored as 0 where there was none; scored as 1 (little) where a TLS occupied an area <10% of the circumference of the tumor; scored as 2 (moderate) when 10–50%; or scored as 3 (abundant) when >50%) [43], histological subtype and grade, tumor size, pT stage, pN stage (or ypT and ypN), and lymphovascular invasion were also recorded. All available full sections were evaluated. For subtyping, cancer tissues were stained immunohistochemically with antibodies against ER (diluted 1:200; NCL-L-ER-6F11, Novocastra, Newcastle-upon-Tyne, UK), PR (diluted 1:200; NCL-L-PGR-312, Novocastra), and HER2 (diluted 1:8; 88–4422, Ventana Medical Systems, Tucson, AZ, USA). ER and PR levels were regarded as positive if there was at least 1% tumor nuclei staining [44]. HR^+^ tumors were defined as those with positive ER and/or PR staining. HER2-overexpressing tumors were defined as those with scores of 3+ by immunohistochemistry or by gene amplification by silver *in situ* hybridization [45]. Lymphoid aggregation with vessels showing features of high endothelial venules (plump, cuboidal endothelial cells) with or without germinal centers was considered as TLS. Histological type was defined based on the 2012 WHO classification criteria, and histological grade was assessed using the modified Bloom–Richardson classification [46].

### Isolation and *ex vivo* expansion of TILs

Breast cancer tissue was placed in RPMI 1640 medium and brought to the laboratory within 2 h of surgery. After washing with phosphate-buffered saline (PBS; pH 7.4) containing 1× ZellShield anticontaminant agent (Minerva Biolabs, Berlin, Gerrmany), the tumor tissues were minced into 1-mm-diameter pieces. The minced fragments were plated into a 24-well plate (four fragments/well) containing 2 mL of TIL culture medium (RPMI 1640 medium supplemented with 10% fetal bovine serum (FBS), 1× ZellShield, 1× 2-mercaptoethanol, and 1,000 IU/mL IL-2 (Miltenyi Biotec, Auburn, CA, USA)). Plates were incubated at 37°C for 14 days to allow TILs to extravasate from the tissue fragment and expand. Half of the medium was changed every 2 days, and the cells were expanded into additional wells when the medium turned yellow. After 14 days, TILs were recovered by centrifugation at 1,500 rpm for 5 min after passing the mixture of tissue fragments and TIL through a 40-μm**-**pore nylon mesh strainer, as described previously [23, 27]. TILs were then counted and cryopreserved.

For further rapid expansion of TILs, the standard REP was employed as previously described [29], with a minor change as follows: 5 × 10^4^ TILs were cultured at 37°C in a T25 flask containing 3 mL of 50% RPMI 1640 and 50% AIM-V media supplemented with 10% FBS, 1× ZellShield, and 1,000 IU/mL IL-2 as well as 30 ng/mL human anti-CD3 antibody (OKT3, Miltenyi Biotec) and 1 × 10^7^ irradiated (50 Gy) allogeneic peripheral blood mononuclear cell feeders. On day 4, 3 mL of the same media was added to the flask, and then 3 mL of media was added every 2 days. After 14 days, TILs (post-REP TILs) were collected, counted, and cryopreserved.

### Preparation of fresh TILs directly from tumor tissue and culturing primary cancer cells

The minced breast cancer tissue was incubated for 1 h in digestion buffer (DMEM-F12, 2% FBS, 1% penicillin/streptomycin, 10 μg/mL insulin, and 10 ng/mL epidermal growth factor) supplemented with 1× collagenase/hyaluronidase (Gendepot, Barker, TX, USA). The digested samples were centrifuged at 80 g for 30 s, and the supernatant was passed through a 70-μm-pore nylon mesh strainer; the cells passing through were collected as one component of fresh TILs. The pellet from the digested samples was resuspended in 0.25% trypsin/EDTA and incubated for 3–5 min to release single cells. Once a single cell suspension was obtained, cold Hank’s balanced salt solution containing 2% FBS was added, and cells were recovered by centrifugation at 300 g for 5 min. The cells from the supernatants and the single cells from the pellet were combined to become the total fresh TILs from tumor tissue, counted, and cryopreserved. For the culture of primary cancer cells, only the single cells from the pellet were used.

To establish primary breast cancer cell cultures, the dissociated breast cancer cells were cultured on 100 mm collagen I-coated plates (Corning, Corning, NY, USA) at 37°C as previously described [47] in DMEM/F12 medium containing 2% FBS, 5 ng/mL epidermal growth factor (Invitrogen, Carlsbad, CA, USA), 0.3 μg/mL hydrocortisone (Sigma-Aldrich, St. Louis, MO, USA), 0.5 ng/mL cholera toxin (Sigma-Aldrich), 5 nM 3,3′,5-triiodo-L-thyronine (Sigma-Aldrich), 0.5 nM β-estradiol (Sigma-Aldrich), 5 μM isoproterenol hydrochloride (Sigma-Aldrich), 50 nM ethanolamine (Sigma-Aldrich), 50 nM O-phosphorylethanolamine (Sigma-Aldrich), 1× insulin/transferrin/selenium (Invitrogen), and 1% penicillin/streptomycin until confluent, and sub-cultured at least twice before cryopreservation.

### Derivation of PDX mouse model

The protocols for this study were approved by the International Animal Care and Use Committee of the Laboratory of Animal Research at the Asan Medical Center, Seoul, Republic of Korea. To derive a PDX mouse model implanted with tumor tissues, four tumor fragments (4 mm^3^ each) from each tumor sample were implanted into an inguinal mammary fat pad of 4–6-week-old immunodeficient female NOD.CB17-*Prkdc*^scid^ (*NOD-SCID*, obtained from Koatech Inc., Seoul, Korea) or NOD.*Cg-Prkdcscid Il2rgtm1Wjl/SzJ* (*NSG*, obtained from Jackson Laboratory, Bar Harbor, ME, USA) mice [5]. For stimulating the growth of breast cancer in the PDX mouse, an estradiol pellet (Innovative Research of America, Sarasota, FL, USA) was also implanted under the skin of the upper back, as previously described [5]. Body weight and tumor growth were monitored once a week. When tumors grew up to 1 cm in diameter, they were excised and some fragments were cryopreserved for future analysis, while other fragments were re-transplanted to NOD.CB17-*Prkdc*^scid^ mice for functional assays. Tumor tissues of PDX mice were immunostained for cancer subtyping, and in all cases they showed the same subtypes as those of the implanted human cancer tissue.

### Flow cytometry analysis of stained single cells

To analyze TIL composition, T cell subsets and their memory phenotypes, single cells dissociated from tumor tissues (fresh TILs), 2 week cultured TILs, and post-REP TILs were stained for surface marker expression as previously described [48]. Briefly, the prepared single cells were first Fc blocked with human BD Fc block (BD Biosciences, San Diego, CA, USA) in ice-cold FACS buffer (PBS containing 2% FBS) for 20 min and then surface-stained at 4°C in the dark for 30 min with the following antibodies: PE-Cy7-labeled anti-CD3 (OKT3; BioLegend, San Diego, CA, USA), APC-Cy7-labeled anti-CD8 (HIT8a; BioLegend), FITC-labeled anti-CCR7 (3D12; BD Biosciences), APC-labeled anti-CD45RO (UCHL1; BioLegend), PerCP-Cy5.5-labeled anti-CD62L (DREG-56; BioLegend), FITC- and PE-labeled anti-CD45 (HI30; BioLegend), Pacific blue-labeled anti-CD95 (DX2; BioLegend), PE-labeled CD11b (ICRF44; BioLegend), APC-labeled CD19 (HIB19; BioLegend), and Pacific blue-labeled CD56 (HCD56; BioLegend). To evaluate the expression of MHC I or II on the surface of primary cancer cells, single cells were similarly surface-stained with Pacific blue-labeled anti-MHC I (W6/32; BioLegend) and Alexa fluor 647-labeled anti-MHC II (Tu39; BioLegend). After washing with FACS buffer, cells were fixed with 2% paraformaldehyde, permeabilized with 90% methanol, and stained at 4°C in the dark for 30 min with FITC-labeled anti-cytokeratins (CKs) 7, 8, 18, and 19 (CK3-6H5; Miltenyi Biotec) and PE-Cy7-labeled anti-EpCAM (9c4; BioLegend). Cancer cells are identified as CK^+^ or EpCAM^+^ cells [47, 49]. The stained cells were assessed using a FACSCanto II (BD Biosciences) and analyzed using FlowJo software (Tree Star, Ashland, OR, USA).

### *In vitro* functional test of TILs

To assess the capability of IFNγ production by TILs, 1 × 10^5^ post-REP TILs were stimulated with PMA (32.4 nM, Sigma-Aldrich) and ION (1 μg/mL, Sigma-Aldrich) in 96-well plates for 24 h. To examine the reactivity of the TILs against autologous cancer cells, the TILs were co-cultured for 24 h with 1 × 10^5^ autologous breast cancer cells in 96-well plates at effector:target cell ratios of 1:1, 2:1, and 4:1. To perform MHC I- or MHC II-blocking experiments during the co-culture, autologous cancer cells were treated with anti-MHC I (W6/32, BioLegend, 10 μg/mL) or anti-MHC II (Tu39, BioLegend, 10 μg/mL) antibody for 1 h prior to the co-culture with TILs. After stimulation of TILs with PMA/ION or co-culture, culture supernatants were collected after centrifuging the culture plate at 1,500 rpm for 5 min to exclude cells and debris. The IFNγ level was measured using a human IFN-γ ELISA kit (K0331121, Koma Biotech, Seoul, Korea) according to the manufacturer’s guidelines.

### *In vivo* functional test of TILs in the PDX mouse model

When implanted tumors in the PDX mice were palpable, one group of mice received 1 × 10^7^ post-REP TILs via the tail vein, while the other group received PBS. In addition, to aid engraftment and/or survival of the transferred TILs both groups of mice received intraperitoneal IL-2 (200,000 IU/mice, Miltenyi Biotec) and IL-15 (1 μg/mice; Peprotech, Rocky Hill, NJ, USA) on the same day (D0). Thereafter, IL-2 (200,000 IU/mice) was administered on the following 2 days (*i.e.*, a total of three times) and IL-15 (1 μg/mice) was administered every other day for 8 days (*i.e.*, a total of five times). Tumor growth was then measured two or three times a week using calipers. Tumor volumes (in mm^3^) were calculated using the formula ½ × length × (width)^2^. A minimum of four mice per group was used for each experiment.

### Statistical analysis

All of the statistical analyses were conducted using SPSS statistical software version 20 (SPSSA). The Kruskal–Wallis test, Mann–Whitney *U*-test, and non-paired Student’s *t*-test were used to evaluate the data. All of the tests were two-sided, and *p* values less than 0.05 were considered significant. Data are shown as the median ± range or mean ± standard deviation (SD). Correlations were assessed using Spearman’s rank correlation analysis.

## SUPPLEMENTARY MATERIALS FIGURES AND TABLES





## Abbreviations

ACT: adoptive cell transfer
CAR: chimeric antigen receptor
CK: cytokeratin
ER: estrogen receptor
FACS: fluorescence-activated cell sorter
FBS: fetal bovine serum
HER2: human epidermal growth factor receptor 2
HR: hormone receptor
H&E: hematoxylin and eosin
IFNγ: interferon-γ
ION: ionomycin
NAC: neoadjuvant chemotherapy
PBS: phosphate-buffered saline
PDX: patient-derived xenograft
PMA: phorbol myristate acetate
PR: progesterone receptor
REP: rapid expansion protocol
SD: standard deviation
T_CM_: central memory T cells
T_EM_: effector memory T cells
TIL: tumor-infiltrating lymphocyte
TLS: tertiary lymphoid structure
TNBC: triple-negative breast cancer
T_SCM_: memory stem T cells
