# Supplementary material for: Expansion of tumor-infiltrating lymphocytes and their potential for application as adoptive cell transfer therapy in human breast cancer
Source: Oncotarget. 2017 Dec 6;8(69):113345–59. doi: 10.18632/oncotarget.23007 (PMC5768332; doi:10.18632/oncotarget.23007)
Supplement: Supplementary file 1 [file oncotarget-08-113345-s001.pdf]

Expansion of tumor-infiltrating lymphocytes and their potential for application as adoptive cell transfer therapy in human breast cancer

SUPPLEMENTARY MATERIALS

Supplementary Table 1: Summary of breast cancer patient characteristics

	n (%)
Age	
<50	89 (44.9)
≥50	109 (55.1)
Menopause	
Pre	119 (60.1)
Post	79 (39.9)
Subtype	
$HR^+/HER2^-$	95 (48)
$HR^+/HER2^+$	26 (13.1)
$HR^-/HER2^+$	20 (10.1)
TNBC	57 (28.8)
Neoadjuvant chemotherapy	
No	149 (75.3)
Yes	49 (24.7)
pT/ypT	
1	61 (30.8)
2	116 (58.6)
3	14 (7.1)
4	1 (0.5)
pN/ypN	
0	128 (64.6)
1	49 (24.7)
2	14 (7.1)
3	7 (3.5)
pTNM/ypTNM stage	
I	51 (25.8)
II	111 (56.1)
III	24 (12.1)
IV	1 (0.5)
Histologic grade	
1	1 (0.5)
2	90 (46.5)
3	103 (52)
Lymphovascular invasion	
Negative	119 (60.1)
Positive	78 (39.4)

Supplementary Table 2: Breast cancer tissue characteristics and TILs yielded after 2 week *ex vivo* culture. See Supplementary_Table_2

Supplementary Table 3: Fold expansion after 2 week REP culture

Sample ID *n* = 45	REP fold expansion
BC16009	5200
BC16010	1460
BC16021	4620
BC16022	2180
BC16031	3000
BC16032	7000
BC16033	9800
BC16034	240
BC16040	690
BC16043	3140
BC16056	1030
BC16086	1740
BC16087	1840
BC16092	3800
BC16096	1660
BC16097	1120
BC16110	2368
BC16121	2640
BC16122	2400
BC16123	250
BC16126	211
BC16127	2300
BC16128	1570
BC16138	2520
BC16143	900
BC16144	310
BC16145	1910
BC16147	1000
BC16148	170
BC16149	2180
BC16158	4100
BC16167	310
BC16175	1080
BC16180	1010
BC16186	2933
BC16194	465
BC16201	515
BC16204	2640
BC16208	952
BC16215	234
BC16216	405
BC16218	3640
BC16220	2080
BC16222	754
BC16223	2020

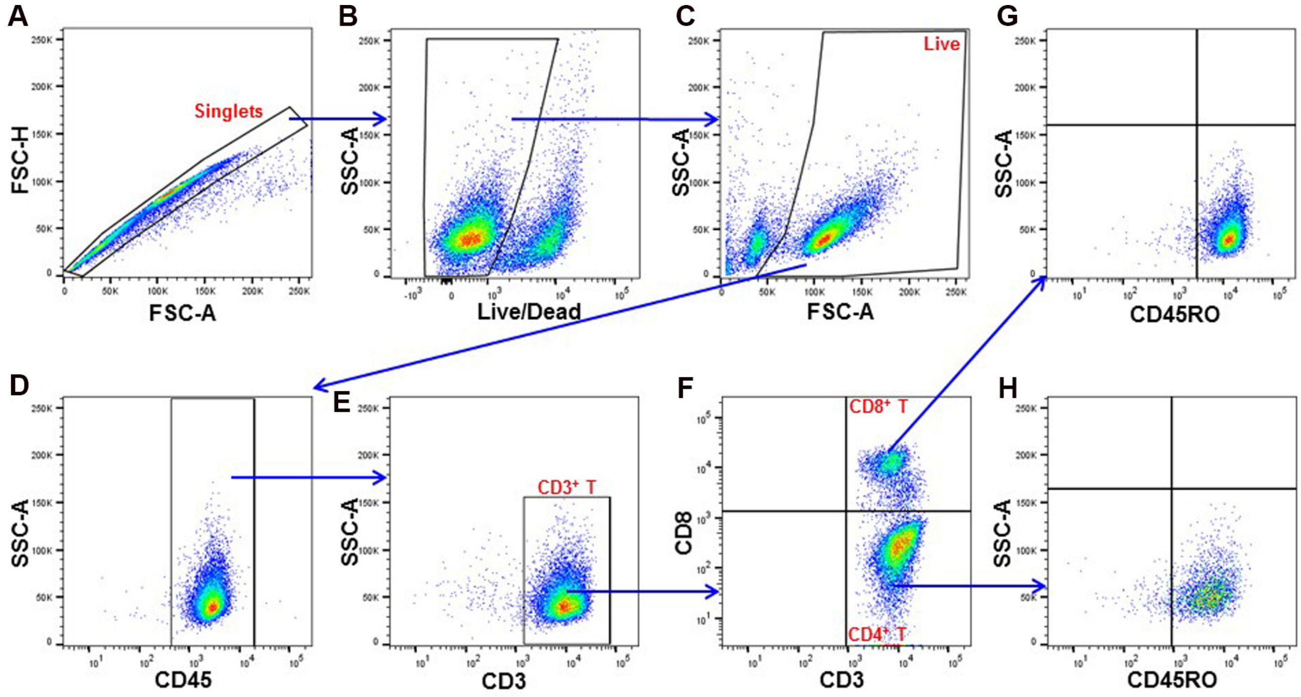

Supplementary Figure 1: Gating strategy used to identify T cell subsets and CD45RO$^+$ memory phenotype T cells. Single cells were stained with Aqua fluorescent reactive dye (dead cell staining), and fluorophore-labeled anti-CD45, -CD3, -CD8, and -CD45RO antibodies and analyzed by flow cytometry. Doublets deviating from the diagonal in the plot of forward scatter area (FSC-A) versus forward scatter height (FSC-H) were excluded (**A**). Dead cells were then excluded by their staining with Aqua fluorescent reactive dye (**B**). Live cells, which are higher up on FSC-A and side scatter area (SSC-A) profile, were selected (**C**); CD45$^+$ hematopoietic cells were then selected (**D**); CD3$^+$ T cells were identified among CD45$^+$ cells (**E**); CD8$^+$ T cells (CD3$^+$CD8$^+$) and CD4$^+$ T cells (CD3$^+$CD8$^-$) were next identified (**F**); CD45RO$^+$ memory phenotype T cells were identified within CD8$^+$ T (**G**) or CD4$^+$ T cells (**H**). Isotype-matched controls for all the antibodies were used to determine positive populations. Identified populations are marked in red text.

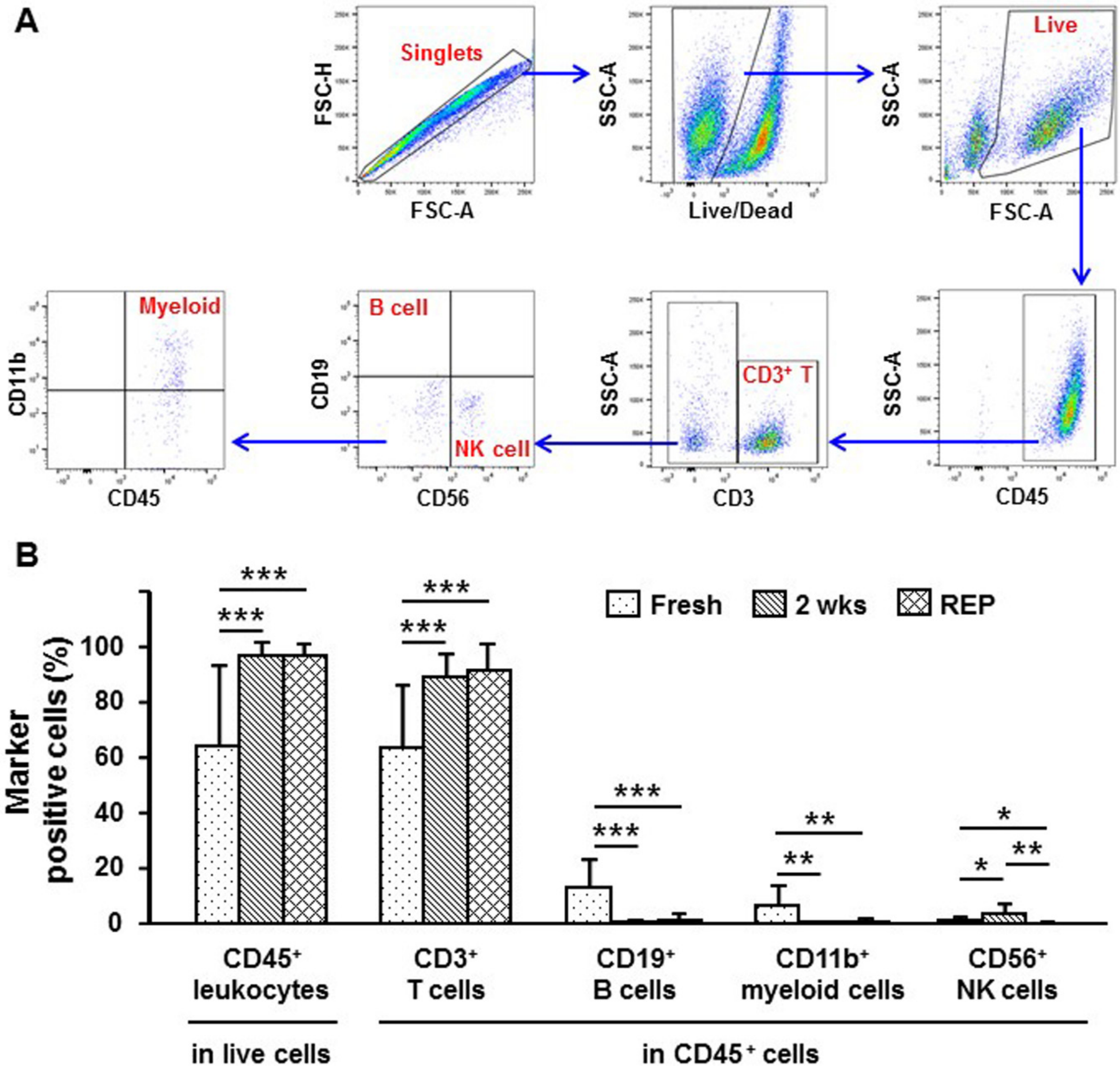

Supplementary Figure 2: Analysis of the composition of major leukocyte subsets in fresh, 2 week, and post-REP TILs. (**A**) Gating strategy used to identify $CD45^+$ hematopoietic cells, $CD3^+$ T cells, $CD19^+$ B cells, $CD56^+$ NK cells, and $CD11b^+$ myeloid cells. Single cells were stained with Aqua fluorescent reactive dye (dead cell staining), and fluorophore-labeled anti-CD45, -CD3, -CD56, -CD19, and -CD11b antibodies, and analyzed by flow cytometry. As described in the Supplementary Figure 1 legend, $CD45^+$ hematopoietic cells were selected, and $CD3^+$ T cells were identified among $CD45^+$ cells, and $CD3^–$ cells were also gated; $CD19^+$ B cells ($CD45^+CD19^+CD56^-$) or $CD56^+$ NK cells ($CD45^+CD19^–CD56^+$) were identified among $CD45^+CD3^–$ cells; $CD11b^+$ myeloid cells were identified among $CD45^+CD3^-CD19^-CD56^-$ cells. Isotype-matched controls for all the antibodies were used to determine positive populations. Identified populations are marked in red text. (**B**) Single cells directly derived from breast cancer tissues (fresh, n = 20), TILs obtained after the 2 week culture of breast cancer tissue fragments (2 wks, n = 20), and post-REP TILs (REP, n = 26) were stained and analyzed by flow cytometry as described above. FACS results are expressed as the mean percentage of positive cells ± SD. Kruskal-Wallis test and Mann-Whitney *U*-test were employed for statistical analysis. $^*p < 0.05$, $^{**}p < 0.01$, $^{***}p < 0.001$.

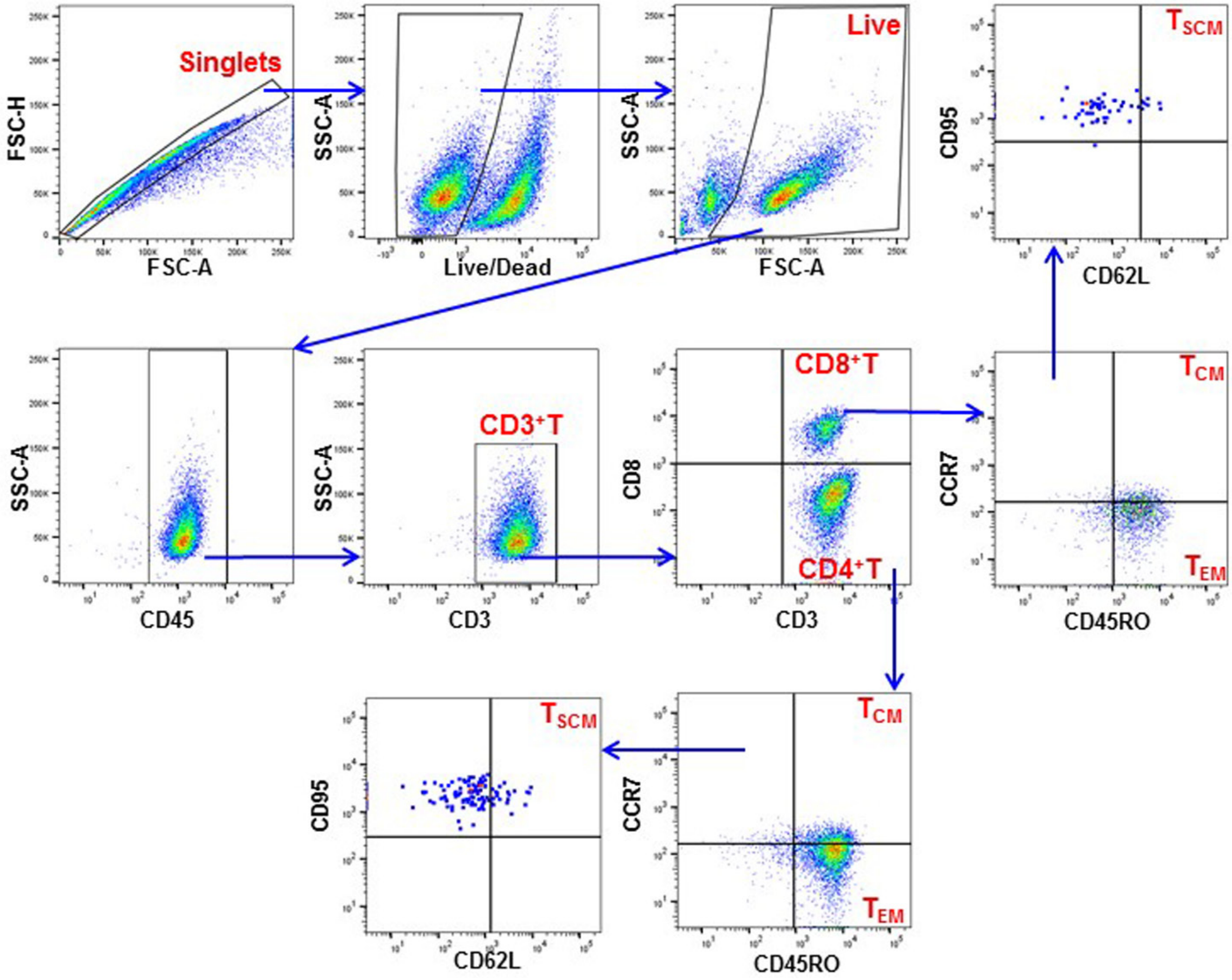

Supplementary Figure 3: Gating strategy used to identify memory T cell subsets within CD8^{+} or CD4^{+} T cells. Single cells were stained with Aqua fluorescent reactive dye (dead cell staining), and fluorophore-labeled anti-CD45, -CD3, -CD8, -CD45RO, -CCR7, -CD62L, and -CD95 antibodies, and analyzed by flow cytometry. As described in the Supplementary Figure 1 legend, CD3^{+} T cells were identified among CD45^{+} cells; CD8^{+} T cells (CD3^{+}CD8^{+}) and CD4^{+} T cells (CD3^{+}CD8^{-}) were then identified; T_{EM} (effector memory T cells defined as CD45RO^{+}CCR7^{-}) and T_{CM} (central memory T cells defined as CD45RO^{+}CCR7^{+}) were identified among CD8^{+} or CD4^{+} T cells; T_{SCM} (memory stem T cells defined as CD45RO^{-}CCR7^{+}CD62L^{+}CD95^{+}) were then identified among CD8^{+}CD45RO^{-}CCR7^{+} or CD4^{+}CD45RO^{-}CCR7^{+} T cells. Isotype-matched controls for all the antibodies were used to determine positive populations. Identified populations are marked in red text.

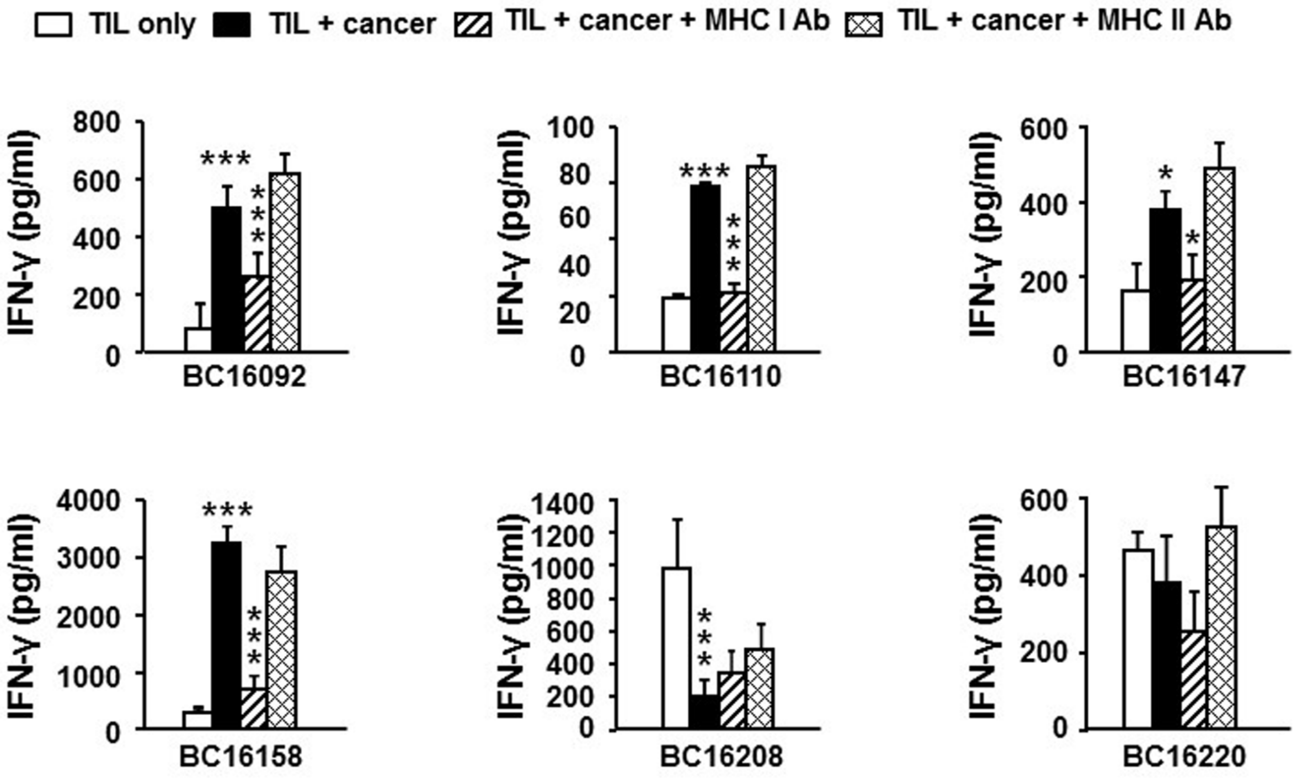

Supplementary Figure 4: Analysis of the reactivity of TILs to autologous primary cancer cells in the presence of MHC blocking antibody. Post-REP TILs derived from each of six patient tissue samples were co-cultured with (TIL + cancer, filled bar) or without (TIL only, open bar) the autologous primary tumor cells (1×10^5) at an effector:target cell ratio of 4:1. Anti-MHC I (TIL + cancer + MHC I Ab, hatched bars) or anti-MHC II (TIL + cancer + MHC II Ab, cross-hatched bars) blocking antibody (Ab) was added 1 h before the co-culture. After 24 h, cell culture supernatants were collected and the IFN**γ** level was measured by ELISA (n =3 or 4 per group). A non-paired Student's t-test was performed between TIL only and TIL+ cancer samples, between TIL + cancer and TIL + cancer + MHC I Ab samples, or between TIL + cancer and TIL + cancer + MHC II Ab samples. $^{*}p < 0.05$, $^{***}p < 0.001$.

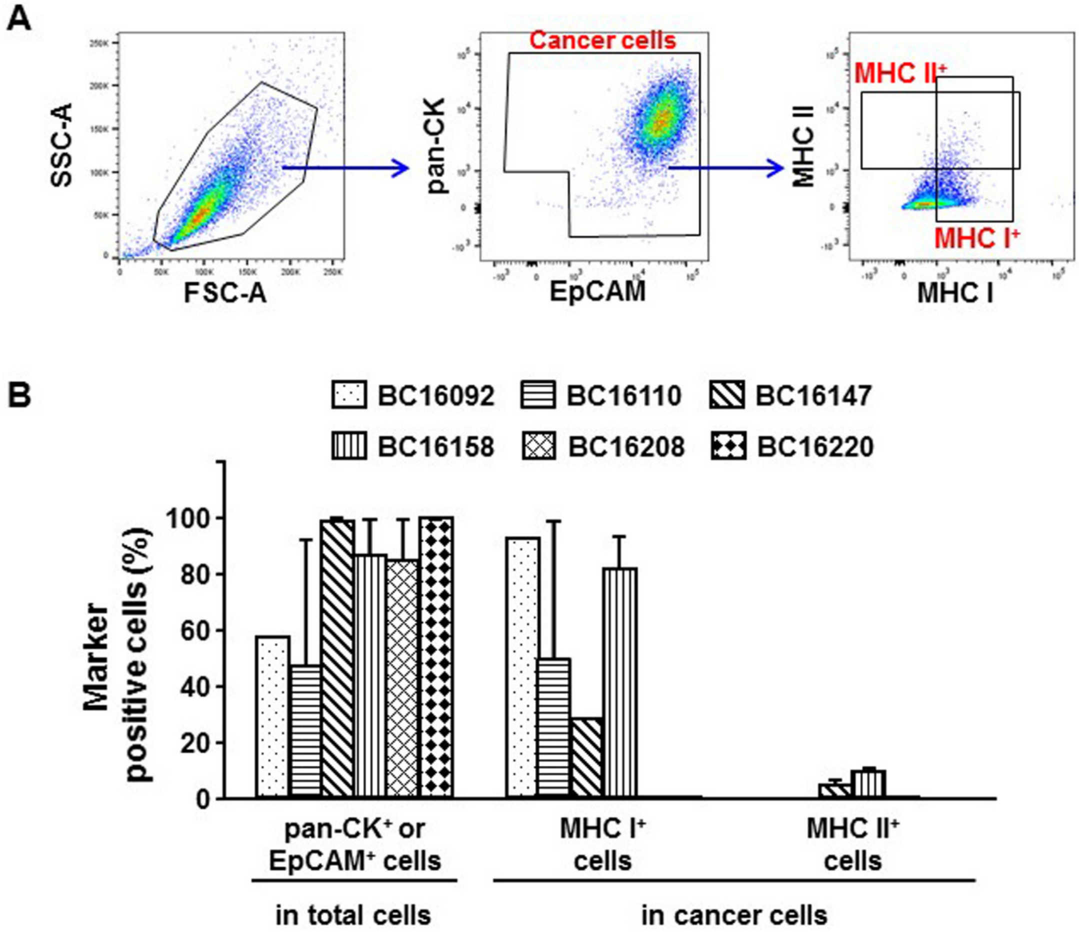

Supplementary Figure 5: The expression level of MHC I or MHC II on primary cancer cells. (**A**) Gating strategy used to identify cancer cells and to measure MHC I or MHC II expression level by flow cytometry. Primary cancer cells were extracellularly stained with fluorophore-labeled anti-pan-cytokeratin (pan-CK), -EpCAM, -MHC I, and -MHC II antibodies and analyzed by flow cytometry. Cells higher up on FSC-A and SSC-A profile were selected; cancer cells (pan-CK$^+$ or EpCAM$^+$) were then selected; MHC I$^+$ or MHC II$^+$ cells were then identified among the cancer cells. Isotype-matched controls for all antibodies were used to determine positive populations. Identified populations are marked in red text. (**B**) The proportion of cancer cells among total live cells, and the proportion of MHC I$^+$ or of MHC II$^+$ cells among the cancer cells, is expressed as the mean ± SD. The first four samples shown left to right on the histogram (BC16092, BC16110, BC16147, and BC16158) are the primary cancer cells that were capable of activating TILs, while the last two samples (BC16208 and BC16220) were not.

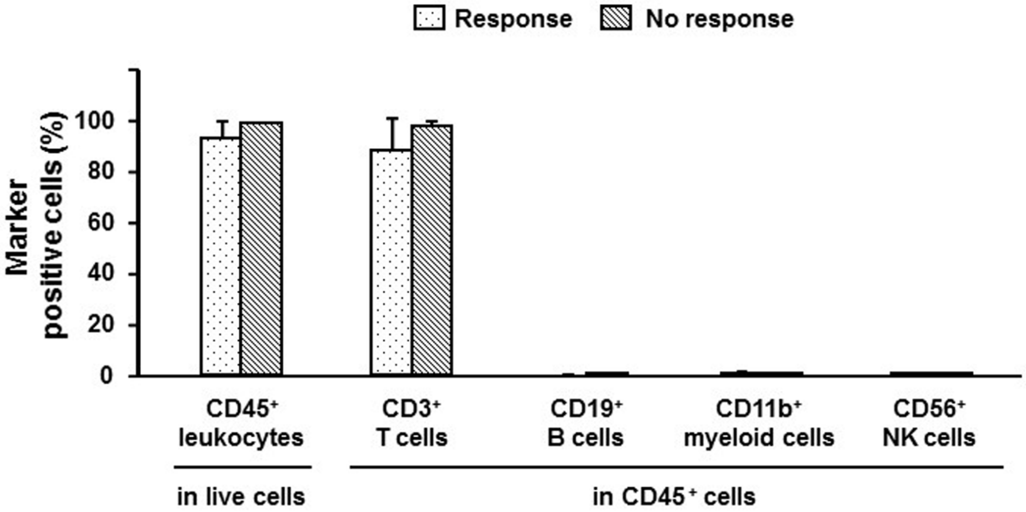

Supplementary Figure 6: Analysis of the composition of major hematopoietic cells in post-REP TILs reactive or non-reactive to autologous cancer cells. Post-REP TILs reactive (n = 4 samples, response) or non-reactive (n = 2 samples, no response) to autologous cancer cells were collected and stained with Aqua fluorescent reactive dye (dead cell staining), and fluorophore-labeled anti-CD45, -CD3, -CD19, -CD56, and -CD11b antibodies, and analyzed by flow cytometry as described in the Supplementary Figure 2 legend. The proportions of $CD45^+$ leukocytes among live cells, and of $CD3^+$ T cells, $CD19^+$ B cells, $CD56^+$ NK cells, and $CD11b^+$ myeloid cells among $CD45^+$ leukocytes, are expressed as the mean ± SD. Kruskal-Wallis test and Mann-Whitney U test were employed for statistical analysis.
